# Supplementary material for: Fecal Microbial Community Composition in Myeloproliferative Neoplasm Patients Is Associated with an Inflammatory State
Source: Microbiol Spectr. 2022 Apr 27;10(3):e00032-22. doi: 10.1128/spectrum.00032-22 (PMC9241690; doi:10.1128/spectrum.00032-22)
Supplement: SUPPLEMENTAL FILE 1 — Supplemental material. Download spectrum.00032-22-s001.pdf, PDF file, 0.9 MB [file spectrum.00032-22-s001.pdf]

## Supplemental Methods

### *Study Participants*

We recruited 25 patients with MPN (essential thrombocythemia (ET), polycythemia vera (PV) and myelofibrosis (MF)) and 25 healthy controls. MPN patients were recruited from the hematology/oncology clinic of UC Irvine Health. Providers identified patients from their panels who fit inclusion criteria based on their clinical history in the electronic medical record. Then, these patients were told about the study, asked additional screening questions to confirm eligibility, and asked to participate. In some cases, spouses or family members of MPN patients were asked to participate as healthy controls. Other healthy controls were recruited through word of mouth from the UC Irvine community. The demographics of the study cohorts, inclusion and exclusion criteria, and disease characteristics are shown in **Supplemental Tables 1-3**. Participants gave their informed consent for this study approved by the Institutional Review Board of the University of California, Irvine.

### *Stool samples*

Participants were given three DNA/RNA shield fecal collection tubes (cat #R1101-E, Zymo Research, Irvine, CA) and three plastic stool collection receptacles to place on the toilet. The fecal collection tubes include a scoop on the lid, participants were instructed to scoop up a pea sized piece of stool, place it in the 9ml of DNA/RNA shield, and mix by inverting 10 times, and keep at room temperature.  Participants were instructed that each sample should be collected from a distinct bowel movement (ideally

on separate days). Participants were not given specific instructions on time of day the sample should be collected, nor given specific instructions on what to eat prior to sample collection. After all samples were collected from the entire cohort DNA was isolated with a ZymoBIOMICS DNA Miniprep Kit according to manufacturer's protocol.

**Microbiome sequencing**

16S rRNA amplicon PCR was performed targeting the full V4 -V5 region using the EMP primers (515F[1] (barcoded) and 926R[2]). The 25 µl PCR reactions were run in a BioRad C1000 PCR machine, (Hercules, CA) with an initial denaturing cycle of 94°C for 3 min and 30 cycles of 94°C, 45 seconds; 55°C, 30 seconds; and 72°C, 60 seconds and the final extension at 72°C for 10 minutes. PCR products were pooled based on visual quantification using 1 µl for strong products, 2 µl for medium products and 3 µl for weak products. The pool of PCR products was cleaned up with Speed Beads (1:1 v/v ratio beads to pool). The beads were pulled down with the magnet and washed twice with 200 µl 80% Ethanol. After drying the bead pellet on the magnet PCR products were released from the beads by adding water. The library was sequenced at the UC Irvine Genomics High Throughput Facility using the Illumina Miseq (v3 chemistry) with a PE300 sequencing length.

The raw sequence data were imported into QIIME2 2018.4[3] and demultiplexed. 12.4 M paired end reads were binned into the designated barcodes (153 samples including two duplicate samples and two positive control mock communities). The mock communities used as positive controls were purchased from Zymo Research (Catalog #D6305). The

average number of demultiplexed reads per sample was 81,081 ± 28,830; the lowest number of reads per sample were 3,782. Sequence quality check, trimming, denoising (DADA2[4] within QIIME2) were carried out, which resulted in 7.9 million paired end reads (averaging 51,651 merged, non-chimeric reads per sample).

The sequences were assigned a taxonomic classification using the May 2013 greengenes database[5] (greengenes.secondgenome.com), trained with the primer pairs that were used to amplify the 16S region.

***Data Availability***

Code used for the statistical analysis can be found on GitHub under the repository: https://github.com/aoliver44/MPN_project. A dockerfile is provided to reproduce the environment and packages necessary for the code. All sequence data can be found under the BioProject PRJNA795185.

***Cytokine Analysis***

Peripheral blood from fifteen MPN individuals and five healthy controls was collected into EDTA tubes and immediately centrifuged, 1 mL of plasma was frozen at -80°C. Measurement of 12 plasma inflammatory cytokines (TNFα, IFNα2, IP-10, GRO, IL-6, IL-8, IL-1a, IL1-b, IL-10, IL-1ra, IL-17a and IFNɣ) with Luminex multiplex technology according to the manufacturer's instructions (MilliporeSigma, Burlington, MA).

***Statistical Analysis***

Alpha diversity was calculated using the Vegan (v.2.5-6)[6] package in R. Permutational multivariate analysis of variance (PERMANOVA) was used to determine whether the microbial community composition was significantly different between metadata factors (i.e. household, health status), also in Vegan. To determine if there were functional differences, based on inferred gene content, between microbiomes of healthy and MPN patients, Phylogenize (v0.92) [7] was used and the results plotted in R using ggPlot2 (v3.3.0)[8]. Distinguishing which microbial taxa or cytokines may be more indicative of MPN or a healthy state was done using a random forest package in R (rfPermute v2.1.7), using the default parameters[9]. We also used the linear discriminant analysis program Lefse[10] to examine differential taxa abundance between MPN and healthy subjects. Integrating microbial taxa (at the genus level) and cytokine data was done using graph-guided fused LASSO (GFLASSO, R package gflasso v0.0.0.9000)[11], which can handle multiple response variable for a given set of predictors (in this case microbial taxa response predicted by cytokine abundances). We also ran Spearman correlations between cytokines and bacterial genera and corrected for multiple comparisons using a false discovery rate cutoff of 0.05. Where appropriate, repeated measures were taken into account by averaging microbiome (i.e., alpha diversity measures) and cytokine measurements within an individual.

**Supplemental Method References**


1. Parada, A. E., Needham, D. M. & Fuhrman, J. A. Every base matters: assessing small subunit rRNA primers for marine microbiomes with mock communities, time

series and global field samples. *Environ. Microbiol.* **18**, 1403–1414 (2016).

2. Quince, C., Lanzen, A., Davenport, R. J. & Turnbaugh, P. J. Removing Noise From Pyrosequenced Amplicons. *BMC Bioinformatics* (2011). doi:10.1186/1471-2105-12-38
3. Bolyen, E. *et al.* QIIME 2: Reproducible, interactive, scalable, and extensible microbiome data science. (2018). doi:10.7287/peerj.preprints.27295v2
4. Callahan, B. J. *et al.* DADA2: High-resolution sample inference from Illumina amplicon data. *Nat. Methods* (2016). doi:10.1038/nmeth.3869
5. DeSantis, T. Z. *et al.* Greengenes, a chimera-checked 16S rRNA gene database and workbench compatible with ARB. *Appl. Environ. Microbiol.* (2006). doi:10.1128/AEM.03006-05
6. Oksanen, J. *et al.* vegan: Community Ecology Package. R package version 2.5-2. *Cran R* (2019).
7. Bradley, P. H. & Pollard, K. S. phylogenize: correcting for phylogeny reveals genes associated with microbial distributions. *Bioinformatics* **36**, 1289–1290 (2020).
8. Wickham, H. *ggplot2 Elegant Graphics for Data Analysis*. *Journal of the Royal Statistical Society: Series A (Statistics in Society)* (2016). doi:10.1007/978-3-319-24277-4
9. Archer, E. rfPermute: Estimate Permutation p-Values for Random Forest Importance Metrics. (2019).
10. Segata, N. *et al.* Metagenomic biomarker discovery and explanation. *Genome Biol.* **12**, R60 (2011).

11. Abreu e Lima, F. *et al.* Unraveling lipid metabolism in maize with time-resolved multi-omics data. *Plant J.* **93**, 1102–1115 (2018).

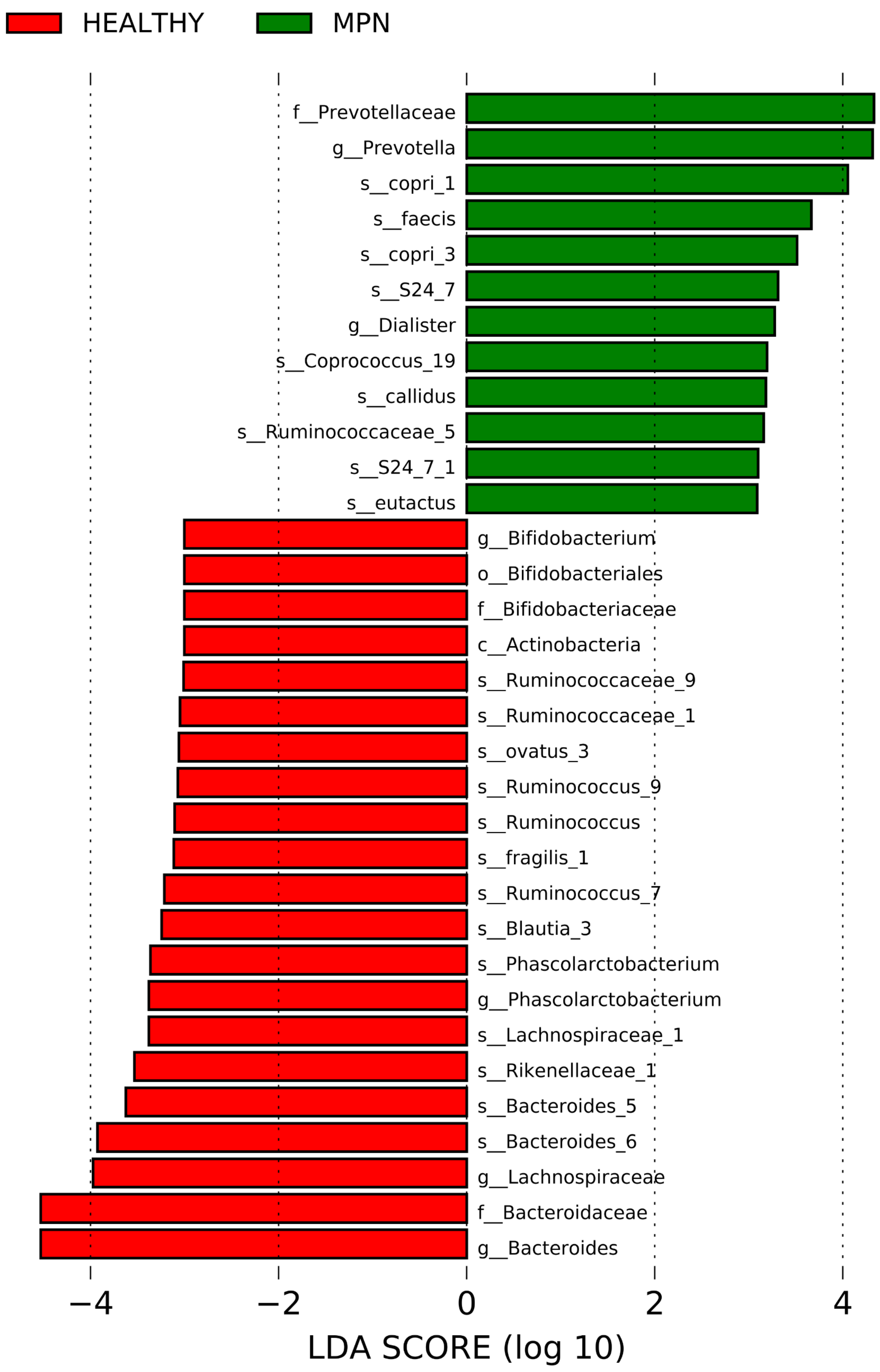


**Supplemental Figure 1**: Lefse (linear discriminant analysis) between healthy and MPN individuals for all microbiome samples.

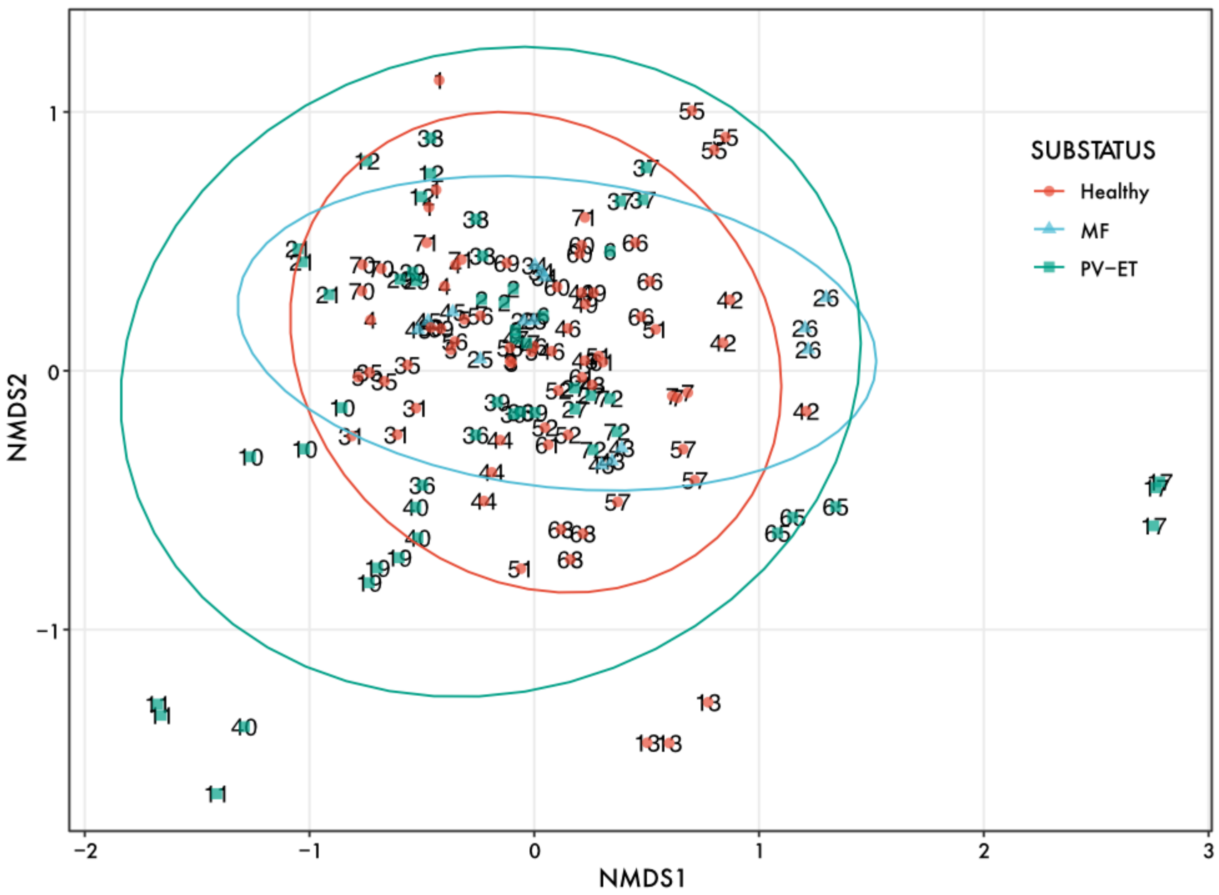


**Supplemental Figure 2:** NMDS ordination of Bray Curtis distances for all microbiome samples. Numbers indicate individual, color and shape represent substatus.

**Supplemental Figure 3:** (A) Spearman correlations between TNFα and microbial genera using all samples from patients with MPN. (B) Scatterplot of between TNFα and Parabacteroides.

A

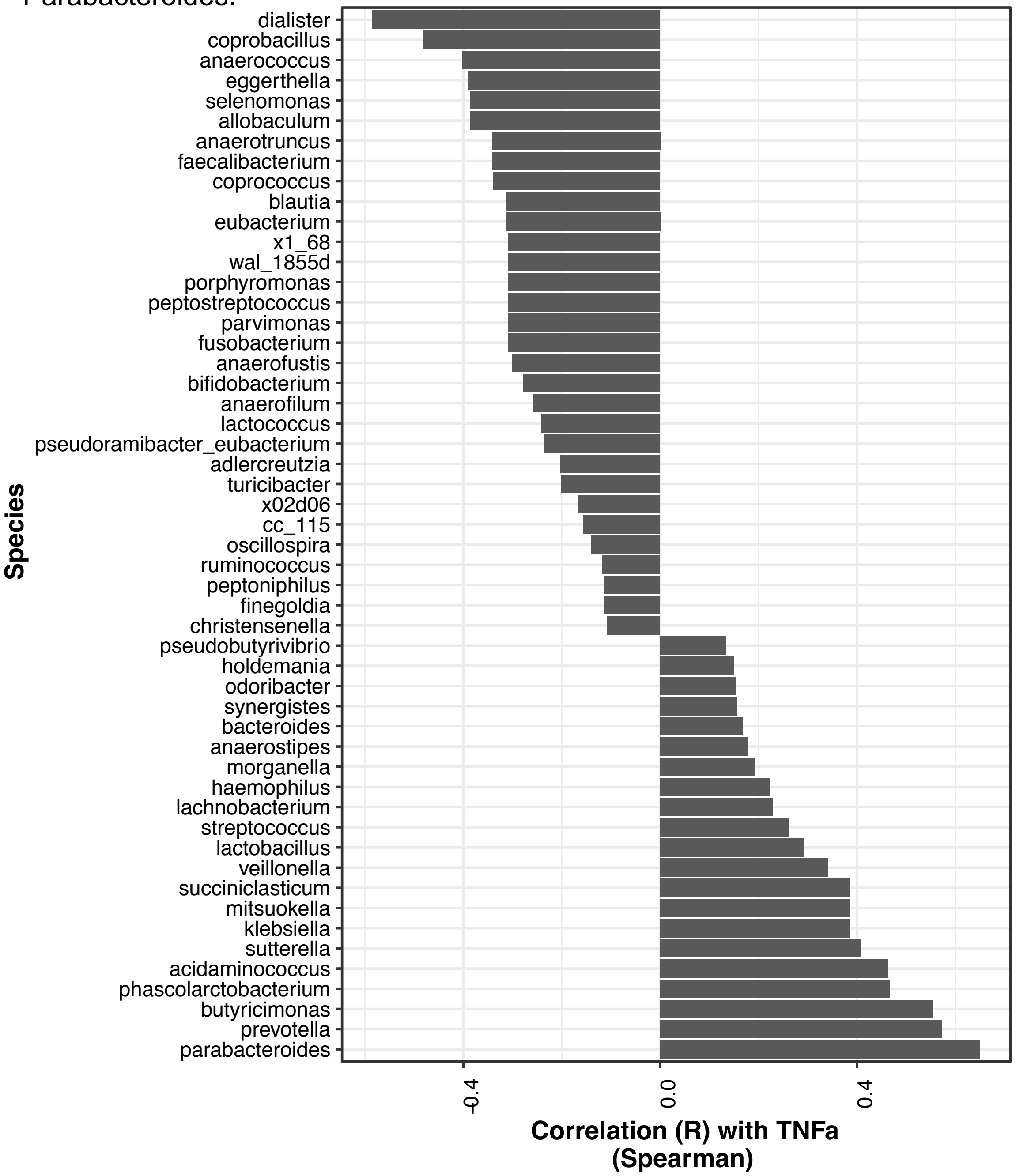


B

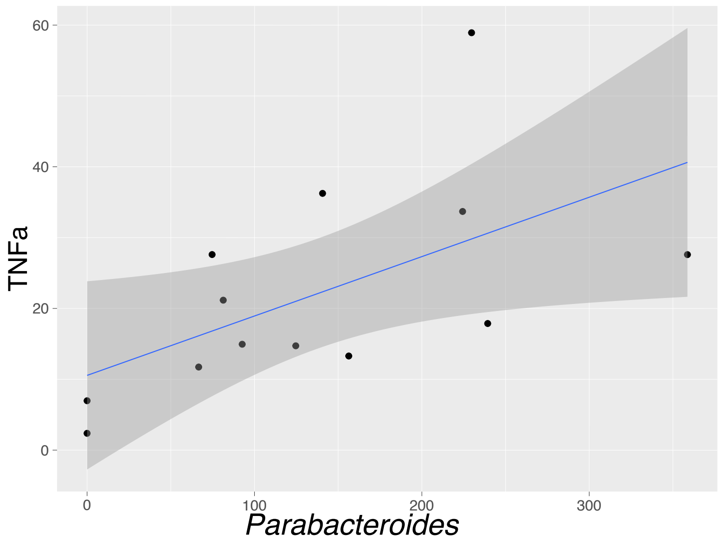

| INCLUSION CRITERIA | EXCLUSION CRITERIA |
|---|---|
| • At least 18 years of age<br>• MPN cohort - Philadelphia chromosome negative MPN including Essential Thrombocythemia (ET), Polycythemia Vera (PV), or Myelofibrosis (MF) according to World Health Organization (WHO) criteria<br>• Health control cohort – no diagnosis of hematologic malignancy | • Gastrointestinal disease (e.g. inflammatory bowel disease, malabsorption, malignancy)<br>• History of pelvic irradiation<br>• Antibiotic use within the past 3 months |

**Supplemental Table 1: Inclusion and Exclusion Criteria**

| Characteristics of subjects | MPN group (N=25) | Non-MPN controls (N=25) |
|---|---|---|
| Age - median (range) | 66 (26-91 years) | 52.5 (26-78 years) |
| Sex - N (%) | | |
| Male | 13 (52%) | 9 (36%) |
| Female | 12 (48%) | 16 (64%) |
| Race - N (%) | | |
| Caucasian | 24 (96%) | 19 (76%) |
| Asian | 1 (4%) | 6 (24%) |
| African American | 0 | 0 |
| Native American | 0 | 0 |
| Ethnicity - N(%) | | |
| Hispanic | 1 (4%) | 1 (4%) |
| Non-Hispanic | 24 (96%) | 24 (4%) |
| Antibiotics exposure - N(%) | 6 (24%) | 1 (4%) |
| Auto-immune disease - N (%) | 6 (24%) | 1 (4%) |
| Family history N (%) | | |
| Hematologic malignancy | 7 (28%) | 3 (16%) |
| MPN | 2 ( 8%) | 3 (16%) |
| Auto-immune disease | 6 (24%) | 3 (16%) |
| Solid cancer | 8 (32%) | 7(28%) |
| Delivery mode | | |
| Vaginal | 23 (92%) | 19 (76%) |
| Cesarean | | |
| Any Supplements | 22 (88%) | 15(65%) |
| Probiotics | 3 (12% ) | 1 (4%) |
| Antioxidants | 2 (8%) | 4 (18%) |
| Specific diet | 9 (36%) | 6 (24%) |
| Breast milk fed at birth | 9 (36%) | 14 (56%) |
| Formula fed | 5 (20%) | 4 (18%) |
| Both | 2 (9%) | 4 (18%) |
| Unknown | 8 (32%) | 2 (9%) |

# Supplemental Table 2: Subject Characteristics

| Characteristic of the disease | MPN Patients (n=25 ) |
|---|---|
| Type of disease | |
| Polycythemia Vera (PV) | 11(44%) |
| Essential Thrombocythemia (ET) | 8 (32%) |
| Myelofibrosis (MF) | 6 (24%) |
| Driver mutation | |
| JAK2V617F | 23 (92%) |
| CALR | 2 (8%) |
| Thrombosis | 5 (20%) |
| Deep Vein Thrombosis | 1 (4%) |
| Stroke | 1 (4%) |
| Myocardial Infarction | 3 (12%) |
| Medication | |
| Aspirin | 22 (88%) |
| Ruxolitinib | 6 (24%) |
| Interferon alpha | 2 (8%) |
| Anagrelide | 1 (4%) |
| Hydroxyurea | 7 (28%) |
| Symptom burden (MPN-SAF score) | |
| High (>20 total score) | 7 (28 %) |
| Low | 18 (72%) |

## Supplemental Table 3. Disease characteristics of the MPN cohort
